# Qingchang Wenzhong Decoction Accelerates Intestinal Mucosal Healing Through Modulation of Dysregulated Gut Microbiome, Intestinal Barrier and Immune Responses in Mice

**DOI:** 10.3389/fphar.2021.738152

**Published:** 2021-09-07

**Authors:** Zhongmei Sun, Junxiang Li, Wenting Wang, Yuyue Liu, Jia Liu, Hui Jiang, Qiongqiong Lu, Panghua Ding, Rui Shi, Xingjie Zhao, Wenjing Yuan, Xiang Tan, Xiaojun Shi, Yunqi Xing, Tangyou Mao

**Affiliations:** ^1^ Department of Gastroenterology, Dongfang Hospital, Beijing University of Chinese Medicine, Beijing, China; ^2^ Graduate School, Beijing University of Chinese Medicine, Beijing, China; ^3^ Department of Traditional Chinese Medicine, Beijing Yangfangdian Hospital, Beijing, China; ^4^ Department of Pathology, Dongfang Hospital, Beijing University of Chinese Medicine, Beijing, China; ^5^ School of Chinese Materia Medica, Beijing University of Chinese Medicine, Beijing, China; ^6^ Dongzhimen Hospital, Beijing University of Chinese Medicine, Beijing, China

**Keywords:** inflammatory bowel disease, Qingchang Wenzhong decoction, mucosal healing, gut microbiota, tryptophan metabolism, intestinal stem cells

## Abstract

Inflammatory bowel disease (IBD), a group of multifactorial and inflammatory infirmities, is closely associated with dysregulation of gut microbiota and host metabolome, but effective treatments are currently limited. Qingchang Wenzhong Decoction (QCWZD) is an effective and classical traditional herbal prescription for the treatment of IBD and has been proved to attenuate intestinal inflammation in a model of acute colitis. However, the role of QCWZD in recovery phase of colitis is unclear. Here, we demonstrated that mice treated with QCWZD showed a faster recovery from dextran sulfate sodium (DSS)-induced epithelial injury, accompanied by reduced mucosal inflammation and attenuated intestinal dysbiosis using bacterial 16S rRNA amplicon sequencing compared to those receiving sterile water. The protective effects of QCWZD are gut microbiota dependent, as demonstrated by fecal microbiome transplantation and antibiotics treatment. Gut microbes transferred from QCWZD-treated mice displayed a similar role in mucosal protection and epithelial regeneration as QCWZD on colitis in mice, and depletion of the gut microbiota through antibiotics treatments diminished the beneficial effects of QCWZD on colitis mice. Moreover, metabolomic analysis revealed metabolic profiles alternations in response to the gut microbiota reprogrammed by QCWZD intervention, especially enhanced tryptophan metabolism, which may further accelerate intestinal stem cells-mediated epithelial regeneration to protect the integrity of intestinal mucosa through activation of Wnt/β-catenin signals. Collectively, our results suggested that orally administrated QCWZD accelerates intestinal mucosal healing through the modulation of dysregulated gut microbiota and metabolism, thus regulating intestinal stem cells-mediated epithelial proliferation, and hold promise for novel microbial-based therapies in the treatment of IBD.

## Introduction

Ulcerative colitis (UC) is one major phenotype of inflammatory bowel disease (IBD), characterized by chronic, continuous, recurrent colonic mucosal inflammation of the colon, affecting the rectum and extending to proximal colon continuously, with typical clinical symptoms including bloody diarrhea, abdominal pain, fecal urgency, and tenesmus (Magro et al., 2017; Feuerstein et al., 2019). The incidence and prevalence of UC has continued to increase throughout the world over past decade, especially in developed countries in North America and Western Europe (Ng et al., 2017). Current therapies including aminosalicylates, corticosteroids, thiopurines, folic acid antagonists, and biological therapies are focused on decreasing intestinal inflammation, inducing and extending disease remission, and controlling complications (Ungaro et al., 2017; Turner et al., 2021). However, these approaches are not curative and have several limitations such as low responsiveness, allergy responses, opportunistic infections, excessive immunosuppression, and refractoriness (Stallmach et al., 2010; Hazel and OʼConnor, 2020). Therefore, novel and safe therapeutic approaches that will improve the clinical phenotype and life quality of UC are expected to be developed.

The human gastrointestinal tract is a complex and dynamic ecosystem harbored trillions of different co-evolved organisms, consisting of bacteria, fungi, viruses, archaea and protozoa (Rooks and Garrett, 2016). Among the earlier studies, the gut microbiome is found to be a pivotal and crucial player in maintaining human health (Wang et al., 2021), which involves in fermentation of dietary components and colonization resistance against pathogens (Cummings and Macfarlane, 1997; Belkaid and Harrison, 2017). Meanwhile, the intestinal commensal bacteria also maintain epithelial barrier integrity, regulate intestinal homeostasis, shapes the mucosal immune system, balances host defense and oral tolerance with microbial metabolites and components, leading to the establishment of host-commensal mutualism (Kayama et al., 2020). Emerging evidence has suggested that the structure, composition and functionality of the intestinal microbiota differs apparently between UC patients and healthy subjects, such as the lower diversity of the gut microbiota, the reduced abundance of beneficial microorganisms (Gilbert et al., 2016), and increased numbers of pathogenic bacteria (Basso et al., 2019). Therefore, targeted regulation of intestinal microbiota may represent a promising strategy in UC control. However, one important and unanswered question is how to modulate intestinal microbial composition and functionality, contributing to enhanced intestinal epithelial proliferation and improved mucosal healing in the treatment of colitis. Despite the microbial-based therapeutic approaches including fecal microbiota transplantation and probiotics have been explored as promising candidates to reprogram microbial balance, epithelial integrity and immune homeostasis, leading to the remission and recovery of colitis (Koretz, 2018; Sood et al., 2021), there is still no consensus on such treatments in UC as a potential clinical strategy, just because of existed challenges such as reliability, safety, and standardization (Narula et al., 2017; Levy and Allegretti, 2019; Cheng and Fischer, 2020).

Complementary and alternative medicines, especially herbal medicines, have increasingly been used in the treatment of UC patients in recent years (Naganuma et al., 2018), and has been shown an indispensable role in regulating the gut microbiota during UC management (Liu et al., 2020; Zou et al., 2020). Qingchang Wenzhong Decoction (QCWZD), an effective and classical herbal prescription for the treatment of UC, consisted with the following herbs as *Coptis*, *Cannon ginger*, *Sophora*, *Indigo naturalis*, *Sanguisorba carbon*, *Aucklandia*, *Pseudo-Ginseng*, and *Licorice*, and has been proved to attenuate intestinal inflammation in a model of acute colitis, which may be related to the regulation of barrier function and intestinal microbiota (Mao et al., 2016; Mao et al., 2017; Sun et al., 2019). However, the role of QCWZD in recovery phase of acute colitis is unclear. In the present study, we demonstrated that mice treated with QCWZD treatment showed a faster recovery from dextran sulfate sodium (DSS)-induced epithelial injury, accompanied by reduced intestinal inflammation and improved integrity of intestinal mucosal barrier. Furthermore, we showed by means of fecal bacterial 16S rRNA sequencing and metabolomic analysis that QCWZD supplement reversed DSS-induced intestinal dysbiosis and altered metabolic profiles, especially enhanced tryptophan metabolism, which further accelerates intestinal stem-cell-mediated epithelial regeneration to protect the integrity of intestinal mucosa through activation of Wnt/β-catenin signals. These findings suggest that QCWZD-induced gut microbiota alterations exert a key protective role in intestinal epithelial regeneration and mucosal repair, and hold promise for novel microbial-based therapies in the treatment of inflammatory bowel disease.

## Materials and Methods

### Preparation and High Performance Liquid Chromatography Analysis of Qingchang Wenzhong Decoction

QCWZD were purchased from the Pharmacy Department of Dongfang Hospital, Beijing University of Chinese Medicine (Beijing, China), consisted with the following herbs as 1.2 g *Coptis*, 2 g *Cannon ginger*, 1.8 g *Sophora*, 0.6 g *Indigo naturalis*, 3 g *Sanguisorba carbon*, 1.2 g *Aucklandia*, 1.2 g *Pseudo-Ginseng*, and 1.2 g *Licorice*. All herbs were extracted using a method of stimulated family decoction, concentrated and dried to form granules. This process was performed by Beijing Tcmages Pharmaceutical Co., Ltd. (Beijing, China) according to Good Manufacturing Practice for Drugs to guarantee the quality. For HPLC analysis, standard of berberine hydrochloride, gallic acid, ginsenoside Rg1, ginsenoside Rb1 and liquiritin were purchased from National Institutes for Food and Drug Control. Then the major components of QCWZD were detected with a Thermo Ultimate 3,000 system and a Symmetry Shield RP18 Column (5 μm, 4.6 mm × 250 mm, 100 Å) was used at a column temperature of 30°C and injection volume was 10 μL. To detect the content of berberine hydrochloride, the sample was eluted with the mobile phase consisted of aqueous solution of acetonitrile and 0.05 mol/L potassium dihydrogen phosphate with a gradient elution at a flow rate of 1.0 ml/min, and the wavelength of the detector was set at 345 nm. Gallic acid was subsequently eluted under the following chromatographic conditions that methanol and 0.05% phosphoric acid with a gradient elution at a flow rate of 1.0 ml/min, and the chromatogram was measured at 272 nm. For ginsenoside Rg1 and ginsenoside Rb1 detection, the mobile phase of A (acetonitrile) and B (water) were optimized and established as following gradient mode: 0–12 min: 81%B, 12–60 min: 81–64%B at a flow rate of 1.0 ml/min with 203 nm detection wavelength. Then, to detect the content of liquiritin the mobile phase of A (acetonitrile) and B (0.05% (v/v) phosphoric acid) under the following gradient program: 81% B for 0–8 min, 81–50% B for 8–35 min, 50–0% B for 35–36 min, 0–81% B for 36–40 min at a flow rate of 1.0 ml/min. The analytes were detected at the wavelength of 237 nm. Afterwards, above major compounds were identified according to the pure standard absorbance spectra and retention time.

### Animals

Six- to eight-week-old female C57BL/6 mice were purchased from SPF Biotechnology Co., Ltd. (Beijing, China). All mice were housed in a specific-pathogen-free facility under artificially controlled conditions (temperature, 20–24°C; relative humidity, 50–60%; lighting cycle, 12/12 h light/dark cycle) and were allowed free access to food and sterile water. All experimental procedures were approved by the Animal Ethics Committee of Beijing University of Chinese Medicine (No. BUCM-4-2020082803–3,149), in accordance with guidelines issued by Regulations of Beijing Laboratory Animal Management.

### Induction of Colitis and Drug Administration

Colitis was induced by administration of 2.5% (w/v) DSS (molecular weight: 36,000–50,000 Da; MP Biomedicals, Santa Ana, CA, United States) ad libitum in drinking water for 7 days followed by 1-week oral treatment of QCWZD at a dose of 1.8 g/kg (DSS + QCWZD group) or sterile water (DSS group). Mice in the Control group were free access to sterile water throughout whole experiment and oral gavage sterile water from Day8. Mice were monitored for clinical parameters including body weight, bleeding, stool consistency and signs of diseases daily.

### Antibiotics Treatment

Six- to eight-week-old female C57BL/6 mice were given 2.5%DSS (w/v) in drinking water for 7 days to induce colitis, then the colitis mice were supplemented with sterile water or QCWZD at 1.8 g/kg in the absence or presence of an antibiotics cocktail (Kanamycin 0.4 mg/ml, Gentamicin 0.035 mg/ml, Metronidazole 0.215 mg/ml, Vancomycin 0.045 mg/ml, Colistin 850 U/mL) (Su et al., 2020) for one week before euthanized with carbon dioxide for colon tissues.

### Fecal Microbiota Transplantation Protocol

The fecal transplantation experiment was performed based on our previous protocol (Sun et al., 2020). Twenty donor mice were randomly divided into four groups including Control group, QCWZD group, DSS group and DSS + QCWZD group. Mice in DSS and DSS + QCWZD group have free access to 2.5% DSS(w/v) 7 days to induce colitis followed by daily gavage of sterile water or 1.8 g/kg QCWZD from Day 8. Meanwhile, mice in Control group and QCWZD group were fed normally and received daily gavage of sterile water or 1.8 g/kg QCWZD days. Fresh fecal material (stool pellets, and cecal and colonic contents) from each mouse were collected 6 times under a laminar flow hood in sterile condition, mixed with phosphate buffered saline (PBS, 200 mg/ml), centrifuged for 60 s at 2000 rpm under 4°C and allowed to settle for 5 min before transfer to recipients. After induction colitis with 2.5%DSS (w/v), recipient mice were randomly assigned into four groups Cont→DSS group, QCWZD→DSS group, DSS→DSS group, and DSS + QCWZD→DSS group, and the fecal supernatants from donors were transplanted into corresponding recipient mice. Clinical status was assessed throughout the experiment and severity of colonic inflammation was scored after euthanized with carbon dioxide on day14.

### Disease Activity Index Analysis

Animals were monitored daily for weight loss, as well as stool consistency and rectal bleeding. The DAI was calculated based on the following parameters: 1) weight loss (0, less than 1%; 1, 1–5%; 2, 5–10%; 3, 10–15%; 4, >15%), 2) stool consistency (0, normal; 2, mushy; 4, diarrhea), and 3) rectal bleeding (0, negative; 2, positive; 4, visible rectal bleeding). The DAI was provided as the average of the scores across the three parameters according to a standard scoring system (Sasaki et al., 2000).

### *In Vivo* Colonic Permeability Assay

Intestinal permeability was examined by oral administration of FITC-dextran (3,000–5,000 kDa, Sigma–Aldrich) as described previously (Chassaing et al., 2014). Briefly, mice were deprived of food and water for 4 h, and blood samples were collected retro-orbitally 2 h after oral inoculation of FITC-dextran (600 mg/kg body weight). Plasma was separated by centrifuging (3,000 rpm, 10 min, 4°C) and analyzed for FTTC-dextran concentration using FLx800 Fluorescence Reader (BioTek, Winooski, VT, United States) (excitation, 485 nm; emission, 520 nm). FITC-dextran concentrations were determined using a standard curve generated by serial dilution of FITC-dextran in mouse serum.

### Histological Analysis

Colonic tissues were fixed in 10% neutral buffered formalin, and then were embedded in paraffin. Then, 5 μm sections were cut and stained with hematoxylin and eosin (H&E) for histological analysis. Scoring for histological changes was performed in a blinded fashion based on inflammatory cell infiltration (0, no infiltration; 1, an increased number of inflammatory cells in the lamina propria; 2, inflammatory cells extending into the submucosa; 3, transmural inflammatory cell infiltration.) and tissue damage (0, no mucosal damage; 1, discrete epithelial lesions; 2, erosions or focal ulcerations; 3, severe mucosal damage with extensive ulceration extending into the bowel wall) as our previously published method (Sun et al., 2020). Scores for inflammatory cell infiltration and epithelial damage were summed, resulting in a total scoring range of 0–6. Additionally, colonic goblet cells were evaluated by alcian blue periodic acid-Schiff (AB/PAS) staining using a Alcian Blue Periodic acid Schiff Stain Kit (Solarbio, Beijing, China) according to the manufacturer’s instructions.

### Immunofluorescence Microscopy

Colonic tissues were frozen in optimal cutting temperature compound (OCT) cryomold in liquid nitrogen. 5μm sections were cut and fixed with ice-cold acetone. The sections were then washed with PBS and blocked with 1% bovine serum albumin (BSA) and Avidin Biotin Blocking Kit (Abcam, Cambridge, England) according to the instructions. Then the tissue sections were incubated with primary antibodies including zonula occludens-1 (ZO-1, 1:200), E-cadherin (1:200) and leucine rich repeat containing G protein-coupled receptor 5 (Lgr5, 1:100). After overnight incubation at 4°C, secondary antibodies were incubated 1 h at room temperature followed by counterstained nuclei using Fluoroshield^™^ with 4′,6-diamidino-2-phenylindole (DAPI) histology mounting medium (Sigma-Aldrich) and examined by immunofluorescence microscopy (Olympus).

### RNA Extraction and Quantitative Reverse Transcription Polymerase Chain Reaction

Total RNA from colon were extracted using Trizol reagent (Invitrogen) according to the manufacturer’s instructions. Then, RT-qPCR reactions were performed with One Step TB Green^®^ PrimeScript^™^ RT-PCR Kit II (Perfect Real Time) (Takara) on 7,300 Real Time PCR System (Applied Biosystems) at 42°C for 5 min, 95°C for 10 s and then cycled 40 times at 95°C for 5 s, 60°C for 31 s, following by melt curve for 15 s at 95°C, 1 min at 60°C, 15 s at 95°C. Relative expression of genes was normalized by glyceraldehyde-3-phosphate dehydrogenase (GAPDH) and determined using the 2^−ΔΔCt^ method. Sequences of primers used in this study are listed in Table 1.

**TABLE 1:** Primer Sequences Used for RT-qPCR.

Target genes	Forward primer (5′–3′)	Reverse primer (5′–3′)
GAPDH	TGG​AAT​CCT​GTG​GCA​TCC​ATG​AAA​C	TAA​AAC​GCA​GCT​CAG​TAA​CAG​TCC​G
IL-1β	CAA​CCA​ACA​AGT​GAT​ATT​CTC​CAT​G	GAT​CCA​CAC​TCT​CCA​GCT​GCA
IL-2	AAA​AGC​TTT​CAA​TTG​GAA​GAT​GCT​G	TTG​AGG​GCT​TGT​TGA​GAT​GA
IL-6	TAG​TCC​TTC​CTA​CCC​CAA​TTT​CC	TTG​GTC​CTT​AGC​CAC​TCC​TTC
IL-10	CCACAAAGCAGCCTTGCA	AGT​AAG​AGC​AGG​CAG​CAT​AGC​A
IL-17	CCA​CGT​CAC​CCT​GGA​CTC​TC	CTCCGCATTGACACAGCG
TNF-α	CCC​TCA​CAC​TCA​GAT​CAT​CTT​CT	GCT​ACG​ACG​TGG​GCT​ACA​G
IFN-γ	TCA​AGT​GGC​ATA​GAT​GTG​GAA​GAA	TGG​CTC​TGC​AGG​ATT​TTC​ATG
ZO-1	CTT​CTC​TTG​CTG​GCC​CTA​AAC	TGG​CTT​CAC​TTG​AGG​TTT​CTG
E-cadherin	CGA​CCG​GAA​GTG​ACT​CGA​AAT	TCA​GAA​CCA​CTG​CCC​TCG​TAA​T
Occludin	AGC​ACT​TAA​CCT​GCC​TGG​AT	AGC​CTG​TGG​AAG​CAA​GAG​AT
Muc1	CTG​TTC​ACC​ACC​ACC​ATG​AC	CTT​GGA​AGG​GCA​AGA​AAA​CC
Muc2	CAA​CAA​GCT​TCA​CCA​CAA​TCT​C	CAG​ACC​AAA​AGC​AGC​AAG​GTA
Lgr5	CCT​ACT​CGA​AGA​CTT​ACC​CAG​T	GCA​TTG​GGG​TGA​ATG​ATA​GCA
Ascl2	AAG​CAC​ACC​TTG​ACT​GGT​ACG	AAG​TGG​ACG​TTT​GCA​CCT​TCA
Wnt3	CTT​CTA​ATG​GAG​CCC​CAC​CT	GAG​GCC​AGA​GAT​GTG​TAC​TGC
Axin2	AAC​CTA​TGC​CCG​TTT​CCT​CTA	GAG​TGT​AAA​GAC​TTG​GTC​CAC​C
Ctnnb1	ATG​GAG​CCG​GAC​AGA​AAA​GC	TGG​GAG​GTG​TCA​ACA​TCT​TCT​T
GSK3β	ACC​GAG​AAC​CAC​CTC​CTT​TG	TCA​CAG​GGA​GTG​TCT​GCT​TG
β-catenin	CGC​TTG​GCT​GAA​CCA​TCA​CA	AGC​AGC​TTT​ATT​AAC​TAC​CAC​CT

### Assay for Myeloperoxidase Activity

MPO activity in the colon was determined using an MPO test kit (Nanjing Jiancheng Bioengineering Institute, Nanjing, China) according to the protocol of the manufacturer.

### Cytokines Quantification by Enzyme-Linked Immunosorbent Assay

Colon tissues were collected for cytokines detection as described previously (Cui, et al., 2018). In brief, 50 mg colon tissues was accurately weighed and homogenized with 450 μL PBS [1:9(w/v)] on ice followed by centrifuged at 3000 rpm for 15 min at 4°C. Then the supernatant was obtained for total protein normalization using a BCA Protein Assay Kit (Solarbio, Beijing) according to the instructions, and the levels of colonic interleukin-6 (IL-6) and tumor necrosis factor-α (TNF-α) were detected using conventional double-sandwich ELISA. The antibodies used for capture and detection were purchased from BD Biosciences. Extinction was analyzed at 450 nm on a Synergy H1 Hybrid Multi-Mode Reader (BioTek, Winooski, VT, United States).

### Fecal Bacterial 16S rRNA Sequencing

The fecal microbiota was examined according to our previously published methods. In brief, genomic DNA was extracted from fecal samples using E.Z.N.A. Soil DNA kit (Omega Biotek, Norcross, GA, United States) according to the manufacturer’s protocols. Then, PCR amplification of the V3∼V4 hypervariable regions of the bacterial 16S rRNA gene was performed with the primers 338F/806R (5′-ACTCCTACGGGAGGCAGCAG-3′’/5′-GGACTACHVGGGTWTCTAAT-3′) at thermocycler PCR system (GeneAmp 9700, Applied Biosystems, Carlsbad, CA, United States). The amplicons were purified with the AxyPrep DNA Gel Extraction Kit (Axygen Biosciences, Union City, CA, United States). Library preparation was performed according to the NEXTflex Rapid DNA-Seq Kit (Bioo Scientific, Austin, TX, United States). The library was pooled in equal molar concentrations and sequenced on an Illumina MiSeq platform. Raw reads were filtered according to read length and quality and then merged. Next, sequences were clustered into operational taxonomic units (OUTs) at a 97% similarity threshold using Usearch (version 7.0) against the SILVA database (version 123) with a confidence threshold of 70%. Alpha diversity estimator calculations were performed using Mothur (version v.1.30.1). A Venn diagram was implemented to show unique and shared OTUs. Principal coordinate analysis (PCoA) was conducted using the representative sequences of OTUs for each sample according to the Bray-Curtis distance. The microbial distribution was visualized using R package (Version 2.15.3) based on community composition information at taxonomic levels. The dominant bacterial community difference was determined using Wilcoxon rank-sum test (for two groups) or Kruskal–Wallis H test (for three groups). Moreover, the potential Kyoto Encyclopedia of Genes and Genomes (KEGG) Ortholog functional profiles of microbial communities were predicted with PICRUSt using STAMP (version 2.1.3).

### Liquid Chromatography-Mass Spectrometry Conditions

Untargeted metabolomics was performed in fecal samples as previously reported (Zeng et al., 2020). In brief, 100 mg sample were ground in 2 ml tube and added 0.6 ml 2-chlorophenylalanine (4 ppm) methanol (−20°C), shaken in a vortex for 30 s. Then, 100 mg glass beads were added in and grind the samples by a high-throughput tissue grinder for 90 s at 60 Hz. After ultrasound at room temperature for 10 min, samples were centrifuged at 4°C for 10 min at 14,000 rpm, and the supernatant was filtered through 0.22 μm membrane to obtain the final supernatant for LC-MS. To monitor repeatability and stability of the system, 20 μL sample was extracted and mixed to make quality control samples. Chromatographic analysis was performed in a Thermo Ultimate 3,000 system equipped with an ACQUITY UPLC^®^ HSS T3 (150 × 2.1 mm, 1.8 μm, Waters) column maintained at 40°C and an injection volume of 2 μL. The mobile phase consisted of 0.1% formic acid in water (A) and 0.1% formic acid in acetonitrile (B) in positive model or 5 mM ammonium formate in water (A) and acetonitrile (B) in negative model at a flow rate of 0.25 ml/min. An increasing linear gradient of solvent B was used as follows: 0–1 min, 2% B; 1–9 min, 2–50% B; 9–12 min, 50–98% B; 12–13.5 min, 98% B; 13.5–14 min, 98–2% B; 14–20 min. Mass spectrometer were executed on the Thermo Q Exactive mass spectrometer with the spray voltage of 3.8 kV and −2.5 kV in positive and negative modes, respectively. Sheath gas and auxiliary gas were set at 30 and 10 arbitrary units. The capillary temperature was 325°C. The analyzer scanned over a mass range of 81–1,000 m/z for full scan at a mass resolution of 70,000. Data dependent acquisition (DDA) MS/MS experiments were performed with HCD scan. The normalized collision energy was 30 eV. Metabolites were identified based on public databases, including Human metabolome database (HMDB, http://www.hmdb.ca/), Kyoto Encyclopedia of Genes and Genomes (KEGG, http://www.genome.jp/kegg/). Metabolic alterations among three groups were visualized using partial least squares discrimination analysis (PLS-DA). Differential metabolites were defined as those with the criteria of both variable importance in the projection (VIP) > 1.0 obtained from orthogonal partial least-squares discrimination analysis (OPLS-DA) and *p* values less than 0.05.

### Statistical Analysis

All data were expressed as means ± standard errors of the means (SEM). Statistical differences between two groups were determined using a Two-tailed Student’s t-test. Comparisons of multiple groups were analyzed by one-way analysis of variance (ANOVA) followed by Tukey’s multiple comparison’s test. All statistical were performed using GraphPad Prism 8.0 (Graph Pad Software, La Jolla, CA, United States). Results were considered to be statistically significant with *p* < 0.05.

## Results

### Major Compounds of Qingchang Wenzhong Decoction by HPLC Analysis

Standard ingredients were selected for herbal medicine of QCWZD according to the Pharmacopoeia of the People’s Republic of China. Subsequently, berberine hydrochloride, gallic acid, ginsenoside Rg1, ginsenoside Rb1 and liquiritin in QCWZD were identified by comparing the retention time and UV spectra of reference standards. To be specific, the contents of the above major active ingredients were 4.6455 ± 0.0048, 0.7178 ± 0.0064, 8.3506 ± 0.0290, 6.2884 ± 0.0656, 0.9661 ± 0.0134 mg/g in QCWZD, respectively, indicating that the major components of QCWZD were stable and repeatable. Chromatogram and chemical structure of the major components involved in QCWZD were depicted in Supplementary Figure S1.

### Administration of Qingchang Wenzhong Decoction Significantly Accelerates Intestinal Mucosal Healing After Dextran Sulfate Sodium Damage

**FIGURE 1:**
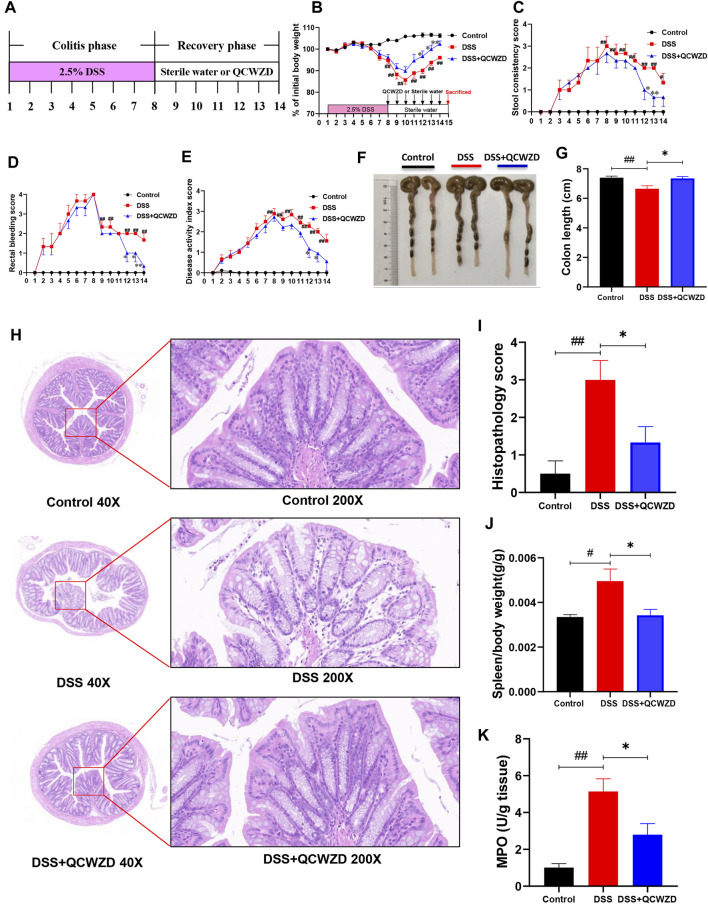
Administration of QCWZD Significantly Accelerates Intestinal Mucosal Healing after DSS Damage. Mice were given 2.5% DSS (w/v) in drinking water for 7 days followed by one week treatment of QCWZD at a dose of 1.8 g/kg or sterile water, then the clinical phenotypes and the severity of colitis were assessed. **(A)** Experimental design, **(B)** Body weight, **(C)** Rectal bleeding, **(D)** Stool consistency, **(E)** Disease activity index (DAI) score, **(F, G)** Colon and colonic length, **(H, I)** Hematoxylin and eosin (H&E) staining of the colon and Histological score. **(K)** MPO activity. The data shown are the mean ± the SEM (*n* = 4–6 mice/group) from one of three experiments performed showing similar results. ^##^ *p* < 0.01, ^#^ *p* < 0.05 versus the Control group; ^∗∗^ *p* < 0.01, ^∗^ *p* < 0.05 versus the DSS group. Significance was determined by one-way ANOVA test (Tukey’s multiple comparison test).

Our previous studies showed that QCWZD is an effective and classical traditional herbal prescription for the treatment of IBD and has been proved to attenuate intestinal inflammation in a model of acute colitis. However, the role of QCWZD in recovery phase of colitis is unclear. To evaluate the effect of QCWZD on intestinal mucosal healing after damage, mice were given 2.5% DSS (w/v) in drinking water for 7 days followed by 1-week treatment of QCWZD at a dose of 1.8 g/kg or sterile water (Figure 1A). In lines with others and our previous studies (Li et al., 2018; Sun et al., 2019), DSS treatment induced severe intestinal inflammation, characterized by developed body weight loss, rectal bleeding and diarrhea, leading to a significantly increased level of disease activity index. While colonic inflammation gradually recovered since the DSS solution was stopped. Interestingly, administration of QCWZD significantly promoted the recovery process, including a faster weight gain, better stool consistency and less rectal bleeding (Figures 1B–E). Moreover, monitoring colon length and histology revealed that DSS-treated mice exhibited significant decrease in colon length, and a delay in restoration of colon epithelial integrity, elimination of inflammatory cells and resolution of colitis when compared to mice in the control group, and those effects were partly relieved by QCWZD treatment (Figures 1F–I). As a major biomarker for phenotyping systemic inflammation, an obvious increase in the spleen-weight ratio was observed in DSS-induced colitis mice, and QCWZD treatment can dramatically relieve the enlargement (Figure 1J). In addition, the results from MPO determination also showed that supplementation of QCWZD significantly decreased MPO activity, an indicator of mucosal neutrophil infiltration, in the colonic mucosa during the recovery phase of acute colitis compared with that of mice with DSS treatment alone (Figure 1K). These results strongly demonstrate that QCWZD significantly accelerate intestinal epithelial wound healing and repair.

### Qingchang Wenzhong Decoction Mitigates Dextran Sulfate Sodium-Induced Immune Responses by Modulating Cytokines Secretion in Mice

**FIGURE 2:**
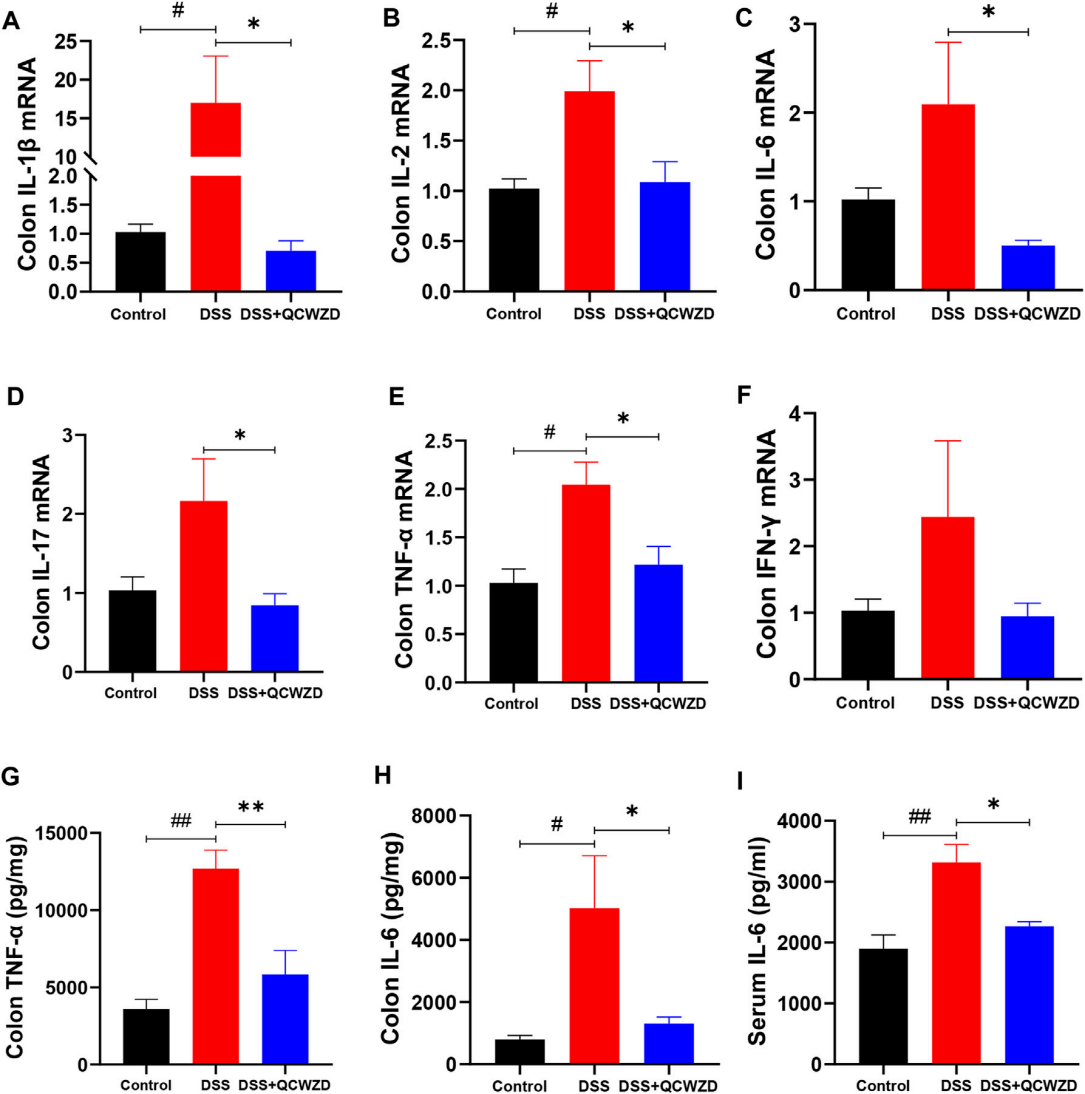
QCWZD Mitigates DSS-Induced Intestinal Injury and Mucosal Inflammation in Mice. Colon and serum samples were isolated from the mice with DSS-induced colitis that had been treated with QCWZD or vehicle control, and inflammatory related cytokines were detected using RT-qPCR and ELISA. RT-qPCR analysis for colonic IL-1β, IL-2, IL-6, IL-17, TNF-α, and IFN-γ **(A–F)**. ELISA analysis for TNF-α and IL-6 in colon and serum **(G–I)**. The data shown are the mean ± the SEM (*n* = 4–6 mice/group) from one of three experiments performed showing similar results. ^##^ *p* < 0.01, ^#^ *p* < 0.05 versus the Control group; ^∗∗^ *p* < 0.01, ^∗^ *p* < 0.05 versus the DSS group. Significance was determined by one-way ANOVA test (Tukey’s multiple comparison test).

To investigate the possibility that QCWZD mitigates murine colitis and intestinal injury by affecting mucosal immunity, we analyzed the mRNA expression of inflammatory related cytokines in colon isolated from the mice with DSS-induced colitis that had been treated with QCWZD or vehicle control. As shown in Figures 2A–F, the levels of major inflammatory cytokines IL-1β, IL-2, IL-6, IL-17, TNF-α, and interferon-γ (IFN-γ) in colon were significantly or relatively higher in the DSS-treated mice than those of the control mice, and QCWZD supplementation alleviated the above increased expressions. Similarly, the results from ELISA showed that QCWZD treatment remarkably inhibited the expressions of IL-6 and TNF-α in colon and serum (Figures 2G–I). Collectively, these data demonstrated that QCWZD mitigates DSS-induced intestinal injury and mucosal inflammation in mice through the inhibition of pro-inflammatory cytokines in colon.

### Supplementation of Qingchang Wenzhong Decoction Modulates Composition and Functionality of Gut Microbiota in Dextran Sulfate Sodium-Induced Colitis Mice

**FIGURE 3:**
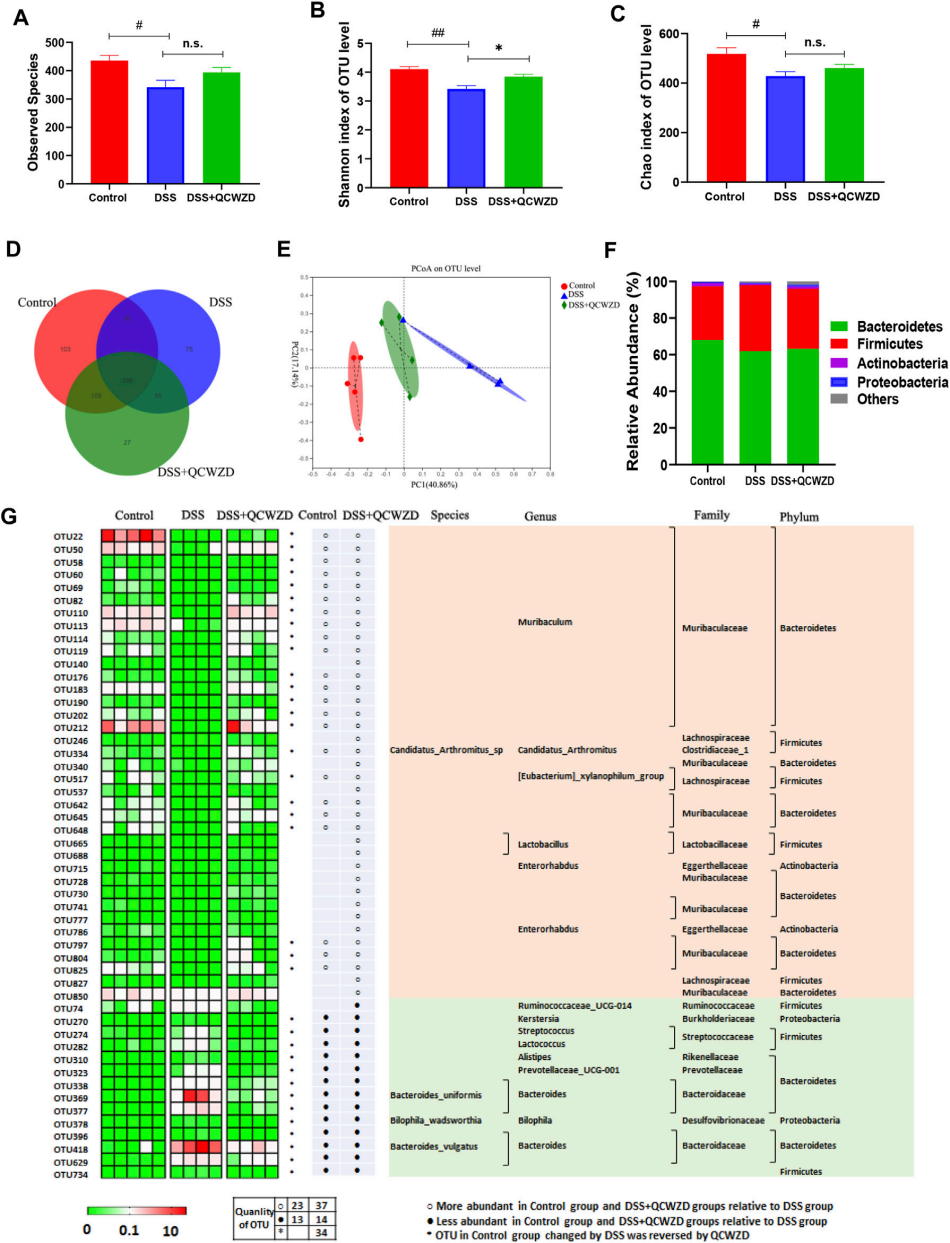
Supplementation of QCWZD Modulates Composition and Functionality of gut microbiota in DSS-Induced Colitis Mice. After a week of QCWZD intervention or sterile water, fresh feces from each mouse were collected for bacterial 16S rRNA sequencing analysis (*n* = 4–6 mice/group). **(A, B)** Comparison of the observed bacterial species and Shannon index of different groups. **(C)** Venn diagram of OTUs. **(D)** Principal Coordinate Analysis (PCoA) based on Bray-Curtis distance among different samples. **(E, F)** Bacterial taxonomic profiling in the phylum and genus level of intestinal bacteria from different mouse groups. **(G)** Heatmap showing the abundance of 51 OTUs significantly altered by QCWZD in DSS-treated mice. The color of the spots in the left panel represents the relative abundance of the OTUs in each group. In the middle panel, white and black circles indicate the OTUs increased or decreased in the Control and DSS + QCWZD groups relative to the DSS group, respectively; black stars represent OTUs whose abundance in the Control mice was altered by DSS treatment and then reversed by QCWZD. The species, genus, family and phylum names of the OTUs are shown on the right panel. The analyses were conducted using R software version 3.3.1.

The commensal microbiota in gut plays a fundamental role in maintaining intestinal homeostasis and human health, and the dysbiosis of gut microbiome has been proved to be closely related to the occurrence and development of colitis. Therefore, fecal samples from mice were used for pyrosequencing-based analysis of bacterial 16S rRNA to evaluate the regulatory effect of QCWZD on the gut microbiota in DSS-induced mice. After removing unqualified sequences, a total of 741,812 raw reads and an average of (57,062 ± 8,512) reads per sample were obtained. The 16S rRNA sequencing results revealed that the overall observed OTUs of intestinal bacteria in DSS-treated mice exhibited a significant decrease compared with the control group, and a relatively higher bacterial species were observed in DSS-induced colitis mice with QCWZD supplement (Figure 3A), although the difference was not statistically significant. We observed a significantly decreased level of Chao index and Shannon index, a measure of the a-diversity of the gut microbiota, in DSS-treated mice compared to the Control group, and while QCWZD treatment reversed the decrease of the diversity to a certain extent (Figures 3B,C). The Venn diagram analyses revealed that 398 OTUs were present in each group, and 448 OTUs coexisted in the Control and DSS groups, 453 OTUs coexisted in the control and DSS + QCWZD groups, and 561 OTUs coexisted in the DSS and DSS + QCWZD groups (Figure 3D), indicating a significant difference among animal groups. Moreover, Bray curtis-based PCoA revealed a distinct clustering of microbiota composition for each treatment, and there is a dramatic separation of microbiota composition between the Control and DSS groups, while the distance between the DSS + QCWZD group and the Control group was closer (Figure 3E). Together, QCWZD had a substantial effect on remodeling the gut microbiome in response to DSS treatment.

To assess the overall composition of the bacterial community in different groups, the degree of bacterial taxonomic similarity were investigated at the different levels. Bacterial taxonomic profiling in the phylum level of intestinal bacteria from different mouse groups showed that DSS-treated mice displayed a relative increase in the abundance of Firmicutes and higher Firmicutes-to-Bacteroidetes ratio compared to mice in the Control group (Figure 3F). We next identified the specific bacterial phylotypes that were altered by QCWZD supplementation. in DSS-treated mice. Compared with the control mice, DSS treatment significantly altered 172 operational taxonomic units (OTUs), producing 14 increased and 158 decreased OTUs (Data not shown). In DSS-treated mice, supplementation with QCWZD increased 37 OTUs and decreased 14 OTUs, resulting in significant changes in a total of 51 distinct OTUs, 36 of which kept the same direction as the control mice. Family analysis revealed that most of these altered OTUs belong to Muribaculaceae (27 OTUs), Lachnospiraceae (6 OTUs), Bacteroidaceae (6 OTUs). Notably, genus-level analysis showed that QCWZD administration enriched the relative abundance of *Lactobacillus* (2 OTUs), and reduced pathogenic species, such as *Bacteroides* (6 OTUs) and *Streptococcus* (Figure 3G). KEGG pathways were further annotated according to the microbiota compositions by PICRUSt analysis to predict bacterial gene functions. Significant differences were observed in 30 KEGG pathways between the DSS and DSS + QCWZD group, and QCWZD supplementation decreased the activities of fructose and mannose metabolism, tyrosine metabolism, pentose phosphate pathway, amino sugar and nucleotide sugar metabolism, and carbohydrate metabolism, while enhanced the activities of energy metabolism, protein folding and associated processing, oxidative phosphorylation, and tryptophan metabolism, which were all induced by DSS treatment (Supplementary Figure S2). Collectively, these results indicate that QCWZD administration modulates the gut microbiota composition and functionality of DSS-treated mice, resulting in a microbiota similar to that of the control mice.

### The Protective Effects of Qingchang Wenzhong Decoction Are Transferable by Fecal Microbiota Transplantation

**FIGURE 4:**
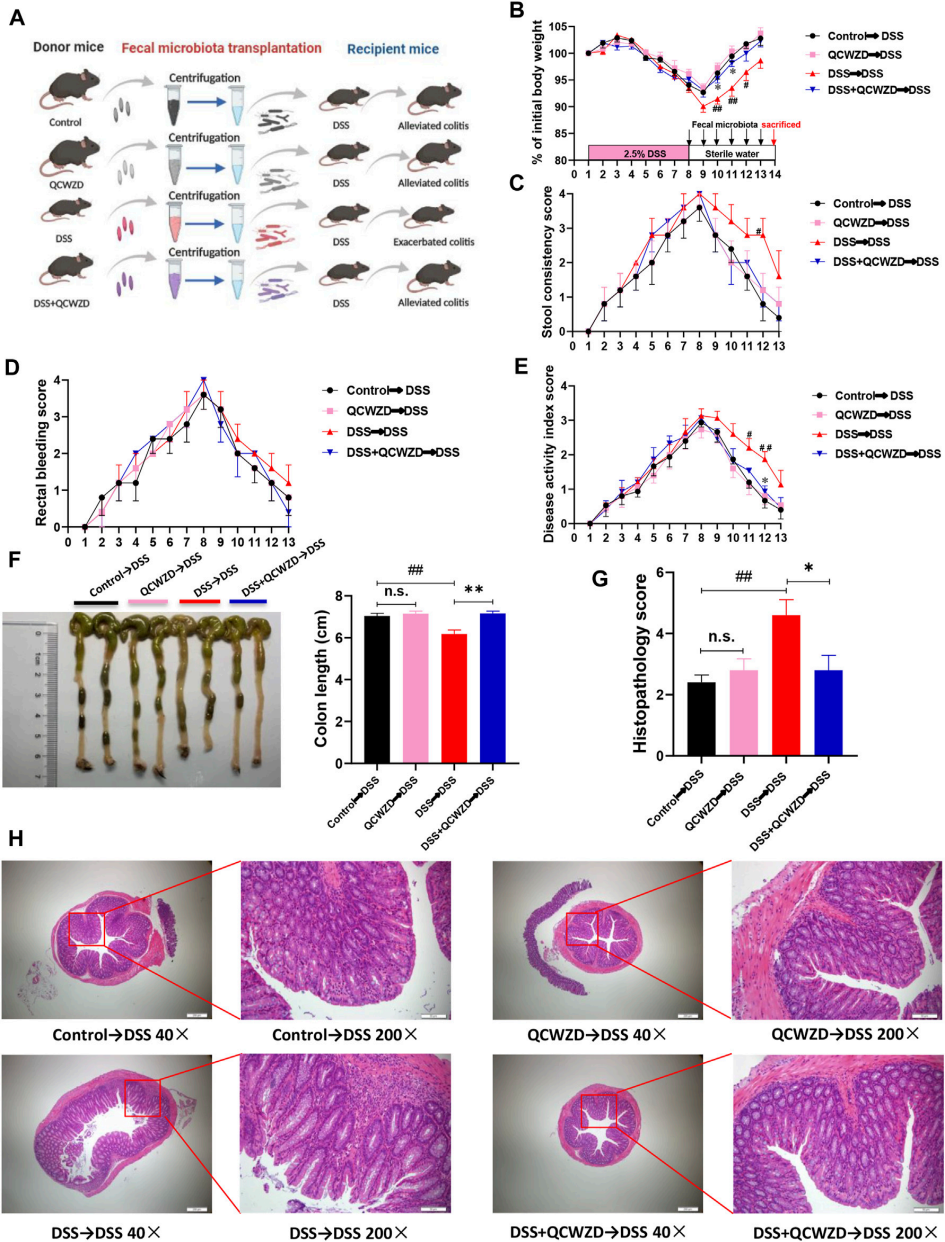
The Protective Effect of QCWZD are Transferable by Fecal Microbiota Transplantation. The feces were collected from the donor mice at the indicated time points and orally gavaged to the corresponding recipients (Control→DSS, QCWZD→DSS, DSS→DSS and DSS+QCWZD→DSS) pretreated with a 7-day DSS solution, then the growth performance and the severity of colitis of all mice were evaluated. **(A)** Study design of fecal transplant experiment. **(B)** Body weight. **(C)** Rectal bleeding. **(D)** stool consistency. **(E)** Disease activity index (DAI) score, **(F, G)** Colon and colonic length, **(H, I)** Hematoxylin and eosin (H&E) staining of the colon and Histological score. **(J)** MPO activity. The data shown are the mean ± the SEM (*n* = 5 mice/group). ^##^ *p* < 0.01, ^#^ *p* < 0.05 versus the Control→DSS group; ^∗∗^ *p* < 0.01, ^∗^ *p* < 0.05 versus the DSS→DSS group. Significance was determined by one-way ANOVA test (Tukey’s multiple comparison test).

To further illustrate the beneficial effects of QCWZD mediated by the gut microbiota and its effects on the intestinal inflammation, we transferred the microbiota of QCWZD-treated mice to DSS-fed mice, followed by examination of colitis-related traits (Figure 4A). We found that the mice that received microbiota from QCWZD-treated donors showed faster recovery process. This is evidenced by increased body weight gain (Figure 4B), reduced fecal consistency score (Figure 4C), and less rectal bleeding (Figure 4D), leading to a lower disease activity index (Figure 4E) compared to DSS-treated recipients of control microbiota, similar to the results shown above. Moreover, macroscopic analysis showed the thinner and longer colons in the recipients of microbiota from QCWZD-treated donors compared to than in the recipients of control microbiota (Figure 4F). Microscopic analysis of colonic tissues of these mice after one week post intervention showed markedly attenuated colitis in recipients that were colonized with QCWZD-induced microbiota, as indicated by relieved cellular infiltration of the lamina propria, relatively intact intestinal mucosa, and decreased inflammatory score (Figures 4G,H). Collectively, these results demonstrate that microbiota transplantation from QCWZD-treated mice trustfully recapitulated the protective effects of QCWZD treatment on DSS-induced colitis, and both QCWZD and QCWZD-treated microbiota could promote the recovery process of intestinal inflammation and accelerate intestinal epithelial wound healing.

### Antibiotics Treatment Diminishes the Beneficial Effects of Qingchang Wenzhong Decoction on Colitis Mice

**FIGURE 5:**
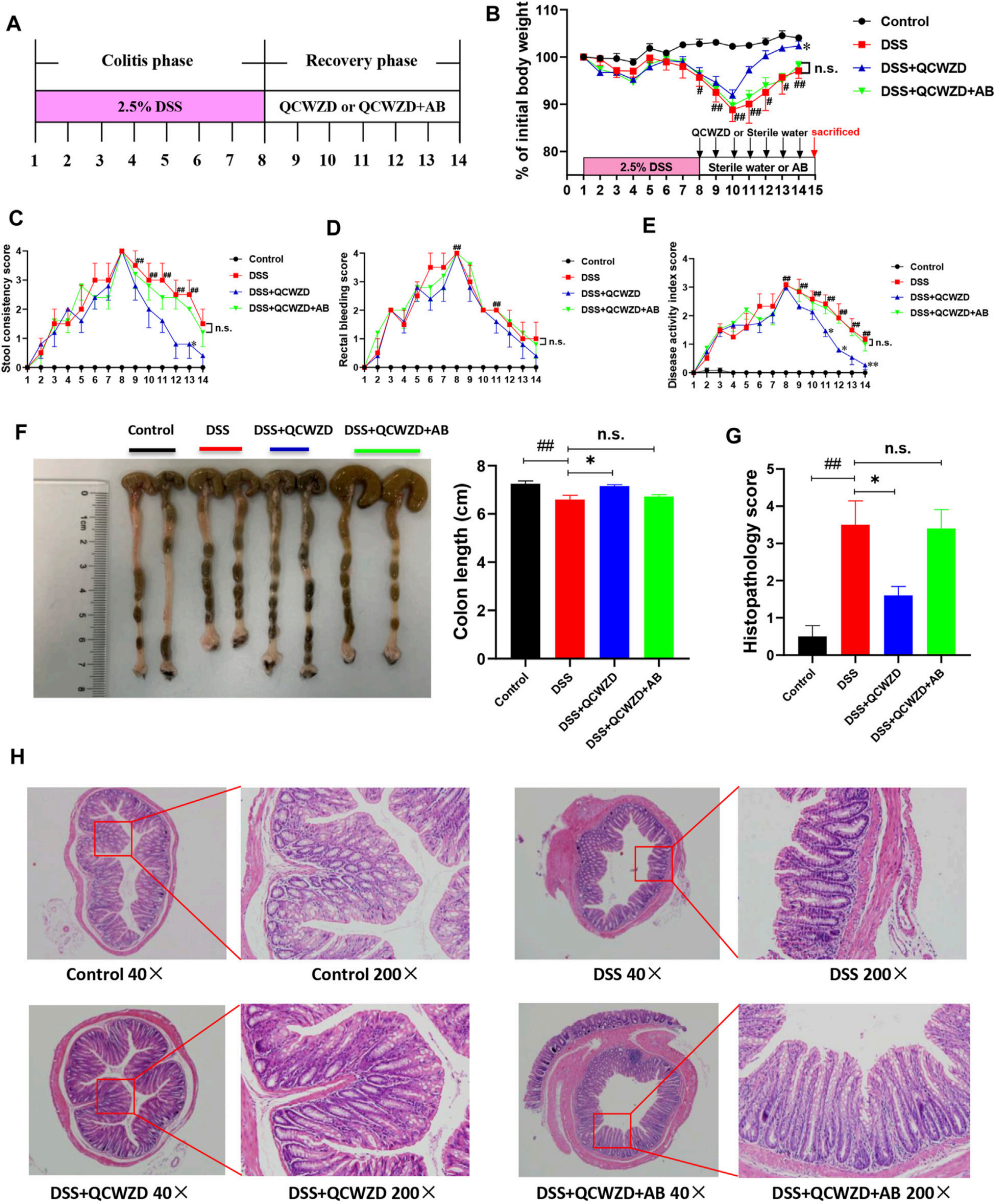
Antibiotics Treatment Diminishes the Beneficial Effects of QCWZD on Colitis Mice. Female mice were given 2.5% DSS (w/v) in drinking water for 7 days followed by one week treatment of QCWZD at a dose of 1.8 g/kg or sterile water in the presence or absence of antibiotics, then the clinical phenotypes were assessed. **(A)** Experimental design, **(B)** Body weight, **(C)** Rectal bleeding, **(D)** Stool consistency, **(E)** Disease activity index score, **(F)** Colon and colonic length, **(G, H)** Hematoxylin and eosin (H&E) staining of the colon and Histological score. The data shown are the mean ± the SEM (*n* = 4–5 mice/group). ^##^ *p* < 0.01, ^#^ *p* < 0.05 versus the Control group; ^∗∗^ *p* < 0.01, ^∗^ *p* < 0.05 versus the DSS group; n. s means no statistical significance. Significance was determined by one-way ANOVA test (Tukey’s multiple comparison test).

To investigate whether the QCWZD-altered gut microbiome was necessary to protect mice against DSS-induced colitis and attenuate inflammation, the colitis mice were treated with a cocktail of antibiotics including Kanamycin, Gentamicin, Metronidazole, Vancomycin and Colistin as shown in Figure 5A. Consistent with the above protective effects on colitis, QCWZD greatly improved clinical symptoms such as body weight gain, rectal bleeding, diarrhea, shortening of colon length and reduced infiltration of inflammatory cells. However, the protective effects of QCWZD on colitis were completely abolished in the present of antibiotics cocktail, as evidenced by no significant difference in the levels of stool consistency score, rectal bleeding score, colon length and histopathological evaluation between the DSS group and QCWZD + AB group (Figures 5B–H), indicating that QCWZD accelerated healing of DSS-induced intestinal mucosal damage in mice in a gut microbiota–dependent manner.

### Metabolic Profiles Are Altered by Qingchang Wenzhong Decoction Treatment in Dextran Sulfate Sodium-Induced Colitis Mice

**FIGURE 6:**
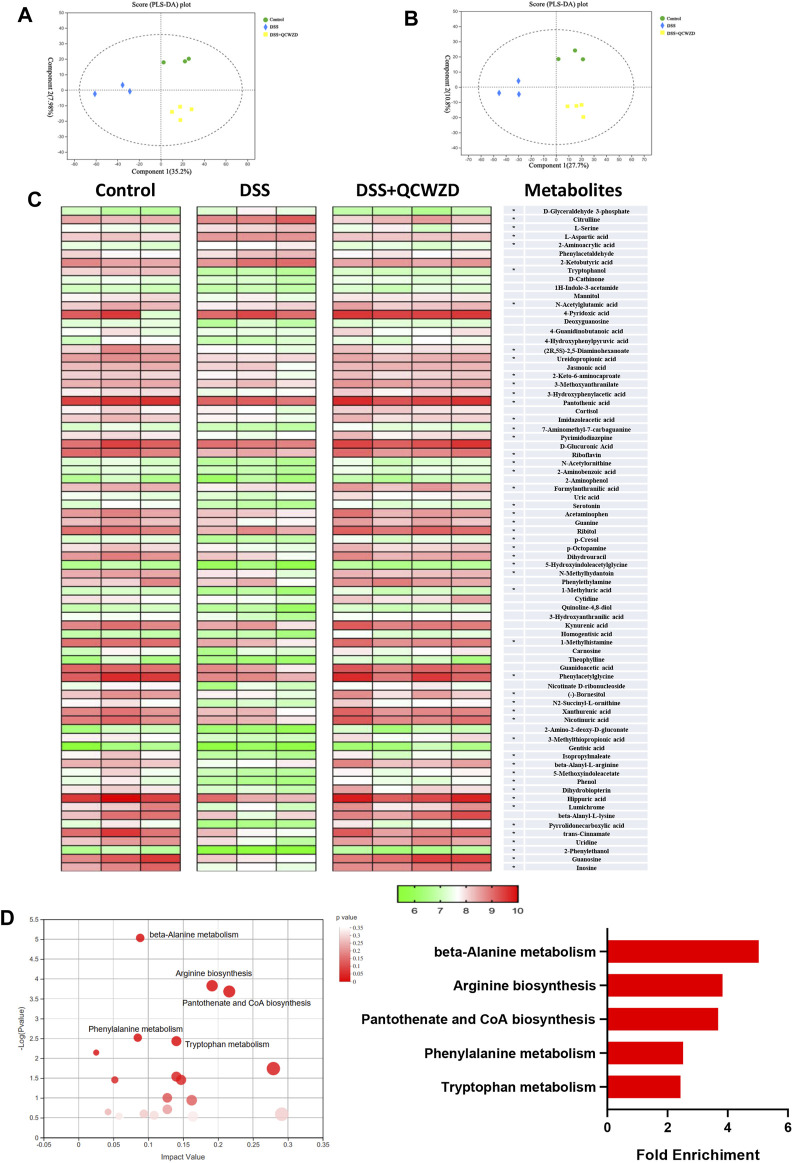
Metabolic Profiles Are Altered by QCWZD Treatment in DSS-Induced Colitis Mice. Fresh fecal samples from each mouse were collected for LC/MS-based untargeted metabolic profiling (*n* = 3–4 mice/group). **(A, B)** PLS-DA plots of the fecal metabolome from all samples in the positive and negative ion modes. **(C)** Heatmaps of the differential metabolites that were altered by DSS feeding compared with QCWZD-fed mice with the double criteria of both VIP >1 and *p* < 0.05. Each column represents an individual sample. Each column represents an individual sample. **(C)** Bubble diagram of metabolic pathways enrichment based on the differential metabolites among three groups. One bubble represents one metabolic pathway. Top 5 pathways were listed on the right.

Emerging studies have suggested that one of the primary modes by which the intestinal microbiome interacts with the host cell is by means of metabolites, which are small molecules that are produced by gut microorganisms through fermenting dietary ingredients (Liu et al., 2017; Ahmadi et al., 2020). Signals from microbial metabolites influence the development and maturation of host immune system, energy metabolism, and maintenance of intestinal homeostasis, and alterations in the metabolite profiles of patients with IBD have been described in previous studies (Lavelle and Sokol, 2020). To further assess metabolic profiles alternations in response to the gut microbiota reprogrammed by QCWZD treatment on DSS-induced colitis mice, LC/MS was subsequently performed to identify the metabolite profiles in feces. PLS-DA analysis showed a distinct segregation among the Control, DSS and DSS + QCWZD groups in both positive and negative model (Figures 6A,B). Compared with the control mice, DSS-induced individuals showed pronounced metabolic alterations in fecal samples, with 7 and 59 metabolites significantly increased and decreased, respectively. Notably, supplementation of QCWZD partially regulated the metabolites altered upon DSS treatment, abrogating 50 of the DSS-induced metabolite changes (5 up-regulated and 45 downregulated; Figure 6C). Next, metabolic pathway analysis affected by QCWZD was carried out based on the 50 differential metabolites and exposed top 5 significantly altered pathways including beta-alanine metabolism, arginine biosynthesis, pantothenate and CoA biosynthesis, phenylalanine metabolism, tryptophan metabolism, which covered 16 differential metabolites including Dihydrouracil, Ureidopropionic acid, Pantothenic acid (included in beta-Alanine metabolism and Pantothenate and CoA biosynthesis), N-Acetylornithine, Citrulline and N-Acetylglutamic (included in Arginine biosynthesis), L-Aspartic acid (included in beta-Alanine metabolism, Arginine biosynthesis, Pantothenate and CoA biosynthesis), beta-Alanyl-L-arginine (included in Arginine biosynthesis), 2-Phenylethanol, trans-Cinnamate, Hippuric acid, Phenylacetylglycine (included in Phenylalanine metabolis), Serotonin, 5-Methoxyindoleacetate, Formylanthranilic acid, 2-Aminobenzoic acid (included in tryptophan metabolism) as shown in Figures 6C,D. It is interesting to note that, QCWZD supplementation significantly inhibited the levels of 5-Methoxyindoleacetate, 5-Hydroxyindoleacetylglycine and 1H-Indole-3-acetamide (Figure 6C), which all included in tryptophan metabolism, indicating a key regulatory role of tryptophan metabolism in the treatment of ulcerative colitis with our herb-based intervention. Taken together, these findings demonstrated that QCWZD was effective in reversing DSS-induced metabolomic disorders, which is related to its effect on modulating the gut microbial dysbiosis.

### Intestinal Stem Cells-Mediated Epithelial Regeneration Promoted by Qingchang Wenzhong Decoction Administration Is Indispensable for Mucosal Healing after Damage

**FIGURE 7:**
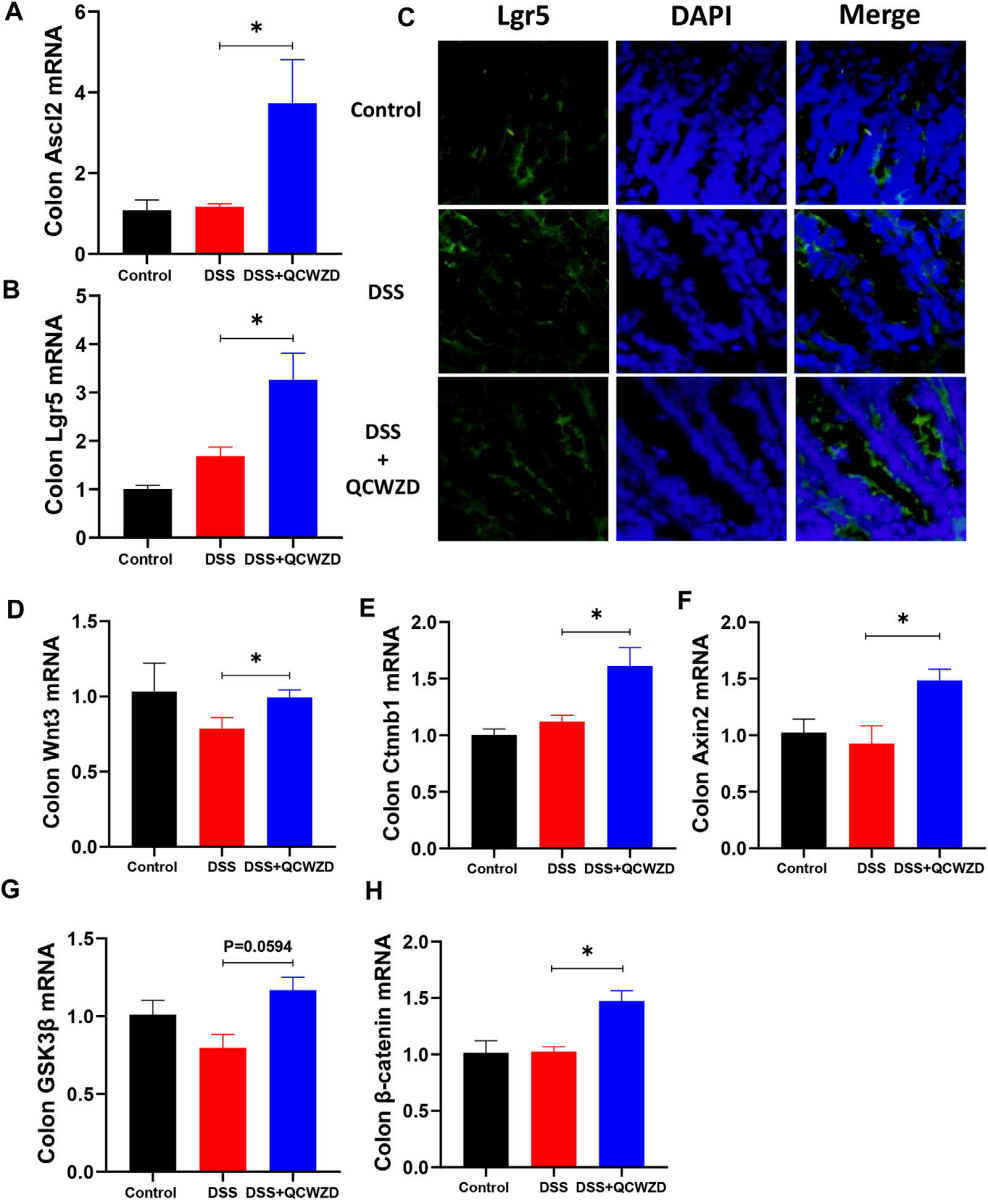
Intestinal Stem Cells-Mediated Epithelial Regeneration Promoted by QCWZD Administration Is Indispensable for Mucosal Healing After Damage. Colon tissues were collected from control or DSS-treated mice after 1-week 1.8 g/kg QCWZD treatment or sterile water for further analysis. **(A, B)** The relative mRNA levels of intestinal stem cells markers (Ascl2 and Lgr5) in colon were determined by RT-qPCR. **(C)** Confocal images (Lgr5 staining, red; and DAPI staining, blue) of the colon with different treatments. **(D–H)** RT-qPCR of relative mRNA expression of Wnt3, Ctnnb1, Axin2, GSK3β, and β-catenin genes of the Wnt/β-catenin axis in colon from each mouse. The data shown are the mean ± the SEM (*n* = 4–6 mice/group) from one of three experiments performed showing similar results. ^∗^ *p* < 0.05 versus the DSS group. Significance was determined by one-way ANOVA test (Tukey’s multiple comparison test).

Given the fact that regulatory effect of QCWZD on microbial tryptophan catabolites, which could affect the proliferation of intestinal stem cells (ISCs) and intestinal epithelium regeneration (Alvarado et al., 2019), we speculated the mucosal healing of intestinal epithelium by QCWZD treatment mostly accounted for intestinal stem cells-mediated epithelial regeneration induced by gut microbiota and metabolism. As shown in Figures 7A,B, administration of QCWZD expedited ISCs proliferation, as evidenced by significant compensated the expressions of stem cell markers such as Lgr5 and achaete-scute like-2 (Ascl2) in colon of colitis mice in the present of QCWZD when compared to mice in DSS group. The stimulatory effect of QCWZD on ISCs was further verified by a higher percentage of Lgr5-positive ISCs in colitis mice with QCWZD treatment compared to those receiving sterile water alone (Figure 7C). We further examined the expression of related genes in Wnt/β-catenin signal pathway, which was the major regulatory mechanism involved in intestinal stem cells renewal and epithelium homeostasis (Kriz and Vladimir, 2018). The results from RT-qPCR analysis showed that expression levels of Wnt/β-catenin pathway-related genes including Wnt Family Member 3 (Wnt3), catenin beta-1 (Ctnnb1), axis inhibition protein 2 (Axin2), glycogen synthase kinase-3β (GSK3β) and β-catenin were significantly enhanced in DSS-treated mice in the presence of QCWZD administration (Figures 7D–H). We, therefore, conclude that altered intestinal microbiota and metabolism response to QCWZD treatment accelerates intestinal stem cells-mediated epithelial regeneration to protect the integrity of intestinal mucosa through activation of Wnt/β-catenin signals.

### Qingchang Wenzhong Decoction Treatment Improves Gut Permeability through Upregulating the Expressions of Tight Junction Proteins and Numbers of Goblet Cells

**FIGURE 8:**
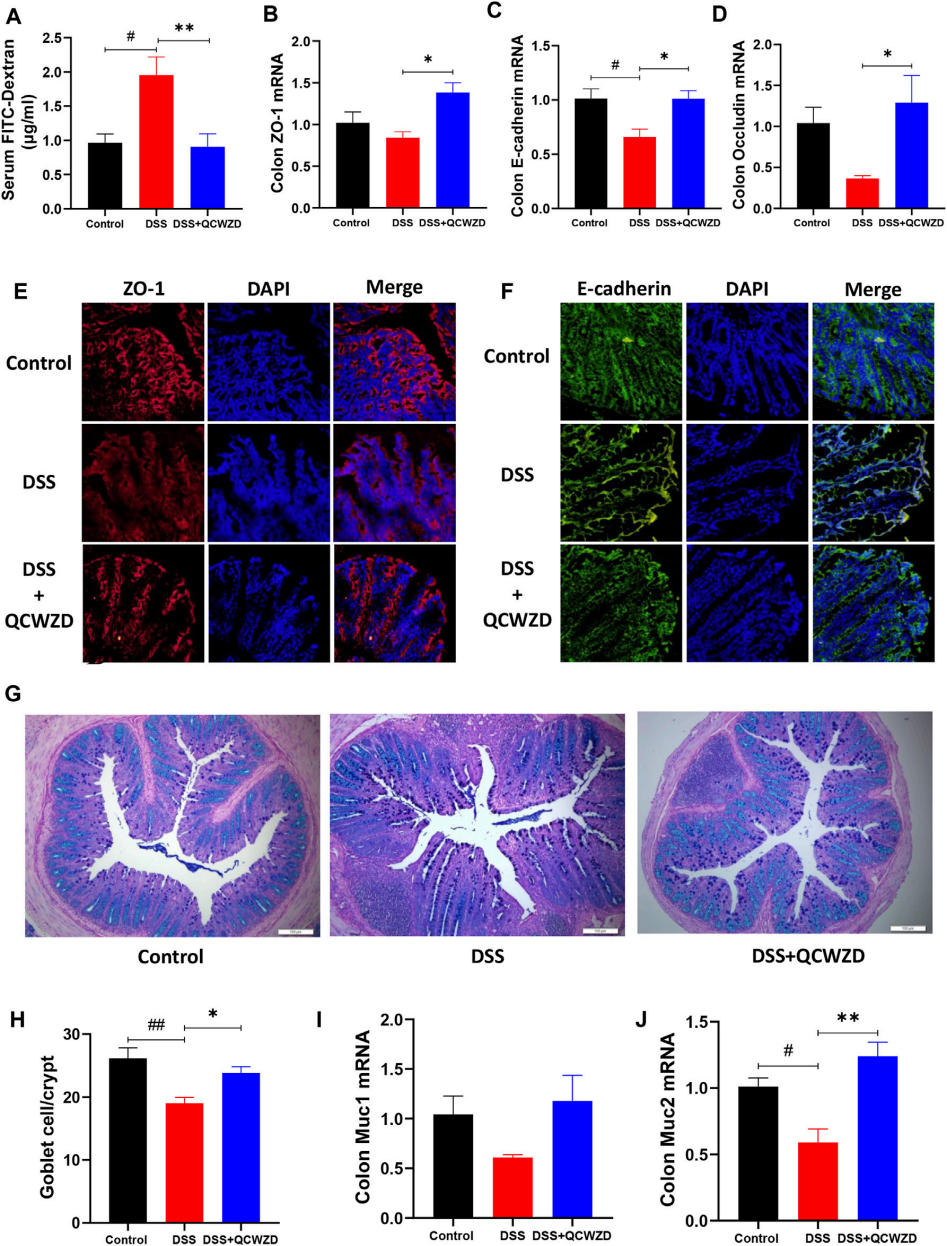
QCWZD Treatment Improves Gut Permeability through Upregulating the Expressions of Tight Junction Proteins and Numbers of Goblet Cells. Healthy or DSS-treated mice were orally administered with 1.8 g/kg QCWZD or sterile water daily, blood samples and colon tissues were collected for further analysis. **(A)** Intestinal barrier functions were assessed in mice by oral gavage of FITC-dextran on day 15, followed by measuring the FITC-dextran signal in blood after 4 h **(B–E)** Colon tissues were collected, and the mRNA levels of ZO-1, E-cadherin and Occludin in colon were detected by RT-qPCR. **(E, F)** Colon tissues were collected in OCT, and frozen sections were prepared and stained by immunofluorescence with anti-ZO-1 or anti-E-cadherin antibodies with Cy3 or FITC-conjugated secondary antibodies. The distribution of ZO-1 and E-cadherin were examined. Representative figures from each group are shown in panels **(E, F)**. **(G, H)** Goblet cells were stained with alcian blue-periodic acid schiff (AB/PAS), and the number of goblet cells per crypt in the colon were quantified. **(I, J)** The mRNA levels of Muc1 and Muc2 in colon were detected by RT-qPCR. The data shown are the mean ± the SEM (*n* = 4–6 mice/group) from one of two experiments performed showing similar results. ^##^ *p* < 0.01, ^#^ *p* < 0.05 versus the Control group; ^∗∗^ *p* < 0.01, ^∗^ *p* < 0.05 versus the DSS group. Significance was determined by one-way ANOVA test (Tukey’s multiple comparison test).

Intrigued by above results, we next examined the impact of QCWZD treatment on gut permeability in DSS-induced mice by oral gavage of FITC-dextran on day 15, followed by measuring the FITC-dextran signal in serum after 2 h. The result showed that the diffusion of FITC-dextran through the epithelium after DSS treatment was significantly increased in DSS-treated mice, and it was compromised in mice with QCWZD supplement (Figure 8A), demonstrating restoration of intestinal barrier functions. To further elucidate the mechanism responsible for the QCWZD-induced alterations in the colonic epithelial barrier function, we examined the expressions of tight junction-associated proteins (ZO-1, E-cadherin and Occludin), which play pivotal roles in establishment and stabilization of intercellular or cellular junctions in epithelial cells. The results from RT-qPCR analysis showed that DSS treatment induced a clear alteration in the mRNA expression level of tight junction complex ZO-1, E-cadherin and Occludin, however, and it was enhanced in DSS-treated colitis mice orally administered with QCWZD (Figures 8B–D). This notion was further confirmed by immunofluorescence microscopy showing a clear increased and normalized expression patterns of ZO-1 and E-cadherin in colonic epithelial cells that were purified from QCWZD-treated colitis mice compared to the levels in epithelial cells from DSS-treated mice (Figures 8E,F). Goblet cells is a kind of mucus secreting cell distributed among the columnar epithelial cells of mucosa and play an important role in maintaining intestinal mucosal barrier and protecting epithelial cells by synthesizing and secreting mucins (Allaire et al., 2018), so we also estimated the numbers of goblet cells in the colon among the three different groups. The results from AB/PAS staining indicated that the DSS-treated mice displayed drastically decreased goblet cells in colons, whereas QCWZD supplementation preserved the goblet cell mass (Figures 8G,H), which corresponds with upregulated Muc1 and Muc2 production (Figures 8I,J). These findings demonstrate that QCWZD treatment upregulated the expressions of tight junction proteins and increased numbers of goblet cells, leading to improve the intestinal permeability and mucosal healing.

### Oral Treatment of Qingchang Wenzhong Decoction Does Not Produce Any Apparent Effects in Chow-Fed Mice

**FIGURE 9:**
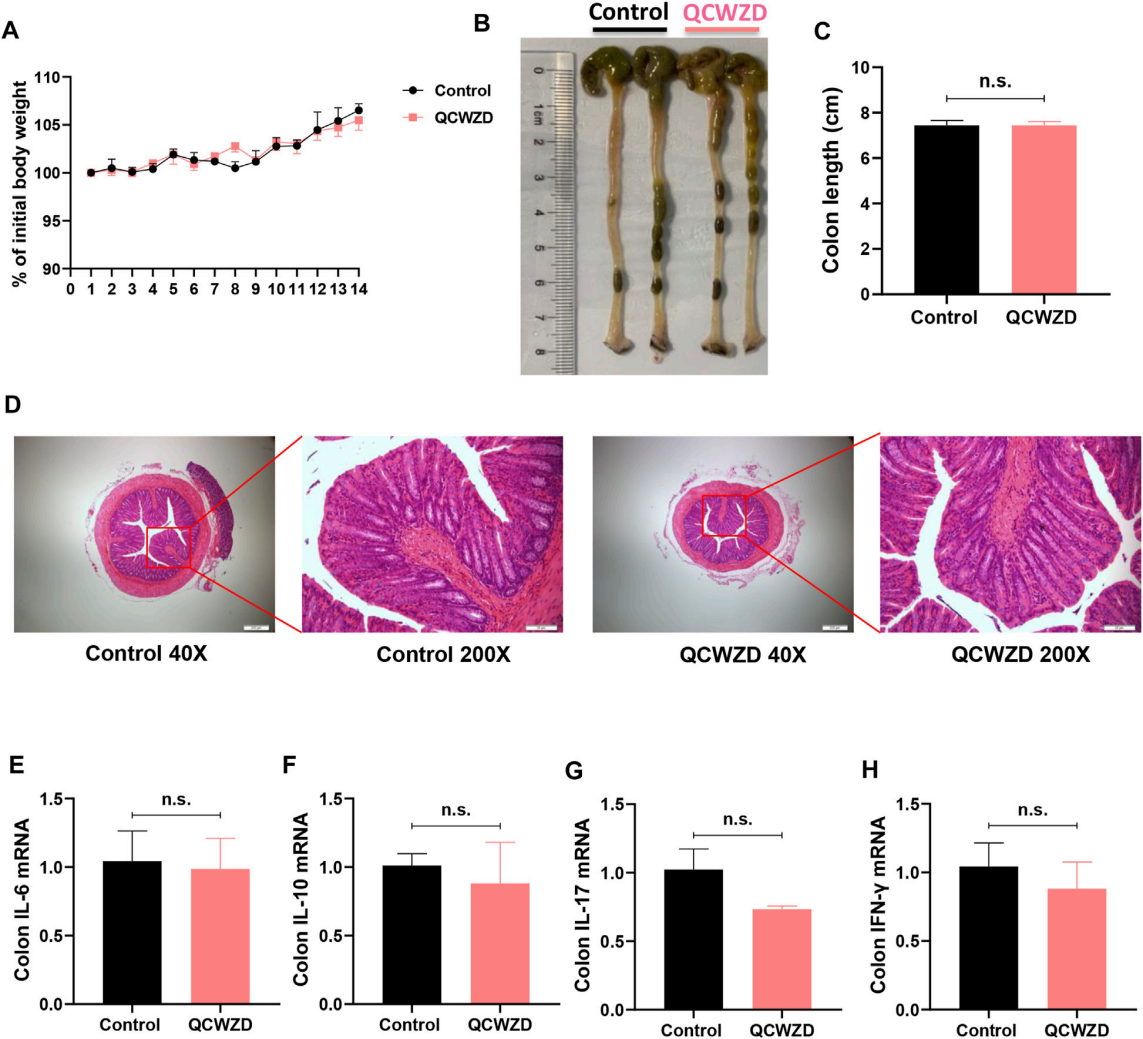
Oral Treatment of QCWZD Does Not Produce Any Apparent Effects in Chow-Fed Mice. Mice were randomly divided into the two groups (*n* = 5). Chow-fed mice were either treated daily with sterile water or 1.8 g/kg/day QCWZD over the course of 14 days. **(A)** Body weight. **(B, C)** Colon length. **(D)** Hematoxylin and eosin **(H&E)** staining of the colon. **(F–I)** RT-qPCR analysis for inflammatory related cytokines IL-6, IL-10, IL-17, and IFN-γ. The data shown are the mean ± the SEM (*n* = 5 mice/group) from one of two experiments performed showing similar results. Significance was determined by a Two-tailed Student’s t-test and ns means no statistical significance between the two groups.

To assess the acute toxicity of QCWZD in animals, ten mice were randomly divided into the two groups. Chow-fed mice were either treated daily with sterile water or 1.8 g/kg/day QCWZD over the course of 14 days. As shown in Figures 9A–D, oral treatment of QCWZD had no apparent effects on body weight gain and intestinal morphology in mice. Moreover, mice with QCWZD supplementation did not show any differences in colonic cytokines related to intestinal inflammation including IL-6, IL-10, IL-17, and IFN-γ compared to the control mice with exposure of sterile water only (Figures 9E–H). Taken together, our data suggest that oral treatment of QCWZD does not produce any apparent effects in mice, and hold promises in clinical treatment of inflammatory bowel disease.

## Discussion

Recent evidence has demonstrated the beneficial effects of microbial-based therapeutic approaches on inflammatory bowel disease, however, how to reestablish the intestinal microecological balance safely and effectively during UC management has become a difficult problem. In the current study, we aimed to seek the answer from traditional Chinese medicine, and our results revealed that Qingchang Wenzhong Decoction treatment resulted in rapid recovery from intestinal inflammation and mucosal damages. We also showed by means of fecal bacterial 16S rRNA sequencing that administration of QCWZD attenuated DSS-induced intestinal dysbiosis. Metabolomic analysis showed metabolic profiles alternations in response to the gut microbiota reprogrammed by QCWZD intervention, especially enhanced tryptophan metabolism, which may further accelerates intestinal stem cells-mediated epithelial regeneration to protect the integrity of intestinal mucosa through activation of Wnt/β-catenin signals. These data highlight the potential of QCWZD as a therapeutic agent for inflammatory bowel disease in terms of its safety and efficacy of modulating gut microbiome, intestinal barrier, immune responses.

Our work reported here demonstrates the promise of a microbial-based therapeutic strategy for ulcerative colitis with orally administered QCWZD. The results from fecal bacterial 16S rRNA sequencing analysis showed that QCWZD significantly attenuated DSS-induced intestinal dysbiosis in mice, especially increased diversity of the gut microbiota and changed the relative abundance of certain bacteria, so we believed that the beneficial effects of QCWZD on intestinal epithelial wound healing and repair are mostly due to remodeling of gut microbiome, which is pivotal in multiple phenotypes associated with inflammatory bowel disease. This notion is supported by the results of fecal microbiota transplantation and antibiotics treatment experiment, which showed that gut microbes transferred from QCWZD-treated mice displayed a similar role in mucosal protection as QCWZD on intestinal inflammation, and antibiotics treatments diminished the beneficial effects of QCWZD on colitis mice. The results from this current study, therefore, provided evidence to demonstrate that QCWZD exert the promotional effects on DSS-induced epithelial injury by altering the structure and functionality of the gut microbiota. Of note, QCWZD intervention increased beneficial microorganism *Lactobacillus,* while selectively depleting pathogenic bacteria *Bacteroides* including species *Bacteroides_vulgatus* and *Bacteroides_uniformis*. *Lactobacillus* is one of probiotic symbiotic bacteria in the gastrointestinal tract of humans and animals, and it persists throughout the life of the host. *Lactobacillus* has been reported to have immunostimulatory property by reducing the pro-inflammatory cytokines, up-regulating SCFAs and restoring the imbalance of gut microbiota (Yang et al., 2021). Moreover, *Lactobacillus* also converts tryptophan to indolealdehyde and indolelactic acid by aromatic amino acid aminotransferase and indole lactate dehydrogenase (Roager and Licht, 2018). *Bacteroides* is a Gram-negative and obligate anaerobic commensal bacterium normally found in the human intestines, and some of its strains such as *B. vulgatus* and *Bacteroides fragilis* are suggested to increase pro-inflammatory cytokine levels, enhance colonic intestinal inflammation and then promote the development of UC (Rabizadeh et al., 2007; Ó Cuív et al., 2017; Ryan et al., 2020). Numerous studies have reported that a higher relative abundance of *Bacteroides* was found in mucosa or stools from mice or patients with inflammatory bowel disease compared with healthy subjects (Sharpton et al., 2017; Chen et al., 2019; Hu et al., 2020; Yan et al., 2020; Chen et al., 2021), which were consistent with our present study, suggesting that it may play an aggravative role in the occurrence and development of UC, and targeted depleting *Bacteroides* by drug or dietary interventions seems to be a promising approach to the treatment of IBD. However, the role of *Bacteroides* in inflammatory bowel disease seems complex, as shown by protection against the development of inflammatory bowel disease in the colitis mice and patients (Ryan et al., 2020; Zhou and Zhi, 2016). It seems controversial with our finding showing that *Bacteroides* was significantly increased both in ulcerative colitis patients and mice. We considered this discrepancy may be related to differences in model system, host genetic background of mice or patients, the type of sample analyzed, or proportion difference of *Bacteroides* species. The context-specific role of *Bacteroides* after QCWZD treatment in regulation of inflammatory bowel disease needs further exploration. Also, further studies are needed to explore the role of other *Bacteroides* species in inflammatory bowel disease.

A growing body of evidence makes it clear that gut microbiota affects the health status of the host through alterations of its metabolic functions (Hong et al., 2020). In the present study, we found the metabolic profiles were altered in colitis mice and the metabolic pathways including beta-alanine metabolism, arginine biosynthesis, pantothenate and CoA biosynthesis, phenylalanine metabolism and tryptophan metabolism were actively responsive to therapeutic interventions of QCWZD. Among them, the most notable changes happened in tryptophan metabolism. Based on the microbiota functional analysis showed that the activities of tryptophan metabolism was significantly decreased by DSS treatment and reversed by QCWZD supplementation, we believed a possible and key regulatory role of tryptophan metabolism in the treatment of ulcerative colitis with our herb-based intervention. The speculation was further verified by untargeted metabolomics showing three microbiota-derived metabolites 5-Methoxyindoleacetate, 5-Hydroxyindoleacetylglycine and 1H-Indole-3-acetamide are differentially changed by QCWZD treatment. These metabolites are included in tryptophan metabolism pathway and belong to indole derivatives, which are ligands for aryl hydrocarbon receptor (AHR). The AHR is widely expressed in intestinal tract, so AhR signaling is considered a crucial component of the intestinal mucosal immunity response at barrier sites by acting on multiple immune cell types, such as intraepithelial lymphocytes, innate lymphoid cells, macrophages, dendritic cells, Th17 cells, and neutrophils (Lamas et al., 2018). In addition to the immunomodulation, commensal bacteria or their metabolites may also activate this receptor, thus contributing to the restoration of gut normobiosis and homeostasis through promotion of epithelial renewal and barrier integrity (Pernomian et al., 2020), indicating a potential target of AHR for the control of mucosal injury and intestinal inflammation, and the development of AHR-based therapies derived from bacteria products may represent an important future alternative for controlling UC. Taken together with our results, these findings demonstrate that QCWZD accelerate intestinal mucosal healing during injury and damage through the modulation of dysregulated gut microbiota and their metabolites, especially microbial tryptophan catabolites.

Our results provide evidence to suggest that one of the potential mechanisms by which QCWZD-altered gut microbiome promote the restoration of epithelial injury may be through the intestinal stem cells-mediated epithelial proliferation. We firstly observed enhanced barrier function in epithelial cells in QCWZD-treated mice with DSS administration, corresponding to decreased epithelial cells damage, and increased numbers of goblet cells, and normalized patterns of tight junction proteins among cells, which were significantly disrupted by the DSS treatment. Given the fact that regulatory effect of QCWZD on microbial tryptophan catabolites, and microbial tryptophan catabolites could affect the proliferation of intestinal stem cells through AHR signaling, we then speculated the modulation of gut microbiota by QCWZD treatment that mostly accounted for the improvement of the gut barrier through the self-renewal and differentiation of intestinal stem cells, which can generate all cell types of mature intestinal epithelium *ex vivo* and *in vivo* (Barker et al., 2007; Sato et al., 2009) and then plays an important role in regeneration of intestinal epithelial under physiological and pathological conditions. This notion was confirmed by the results showing higher expressions of stem cell markers and Wnt/β-catenin pathway-related genes, suggesting that intestinal stem cells-mediated epithelial proliferation was necessary for restoration of epithelial injury after DSS-induced chemical damage, and QCWZD-induced microbiota alteration may be a key mediator for this effect through increased survival of intestinal stem cells.

Taken together, the results from our current study demonstrated that orally administrated QCWZD accelerates intestinal mucosal healing through the modulation of dysregulated gut microbiota and metabolism, thus regulating intestinal stem cells-mediated epithelial proliferation. These findings provide novel insights into the regulatory role of QCWZD in modulating the gut microbiota composition and functionality in epithelium wound healing and repair during intestinal inflammation, and hold promise for novel and safe microbial-based therapies in the treatment of inflammatory bowel disease.

## Data Availability

The raw sequencing data have been deposited in the National Center of Biotechnology Information (NCBI) Sequence Read Archive (SRA) database under the BioProject accession number PRJNA 718383.
