# Supplementary material for: Qingchang Wenzhong Decoction Accelerates Intestinal Mucosal Healing Through Modulation of Dysregulated Gut Microbiome, Intestinal Barrier and Immune Responses in Mice
Source: Front Pharmacol. 2021 Sep 7;12:738152. doi: 10.3389/fphar.2021.738152 (PMC8452913; doi:10.3389/fphar.2021.738152)

**Supplementary Figure S1. Major Compounds of Qingchang Wenzhong Decoction by HPLC Analysis.** Representative HPLC chromatograms of **(A)** *Coptis* **(B)** *Pseudo-Ginseng* **(C)** *Sophora* **(D)** *Licorice* in QCWZD. 1. berberine hydrochloride; 2a. ginsenoside Rg1; 2b. ginsenoside Rg1; 3. gallic acid；4. Liquiritin. The upper one in each figure is standard substances and lower one is QCWZD sample. **(E)** The chemical formula of the active compounds in QCWZD.


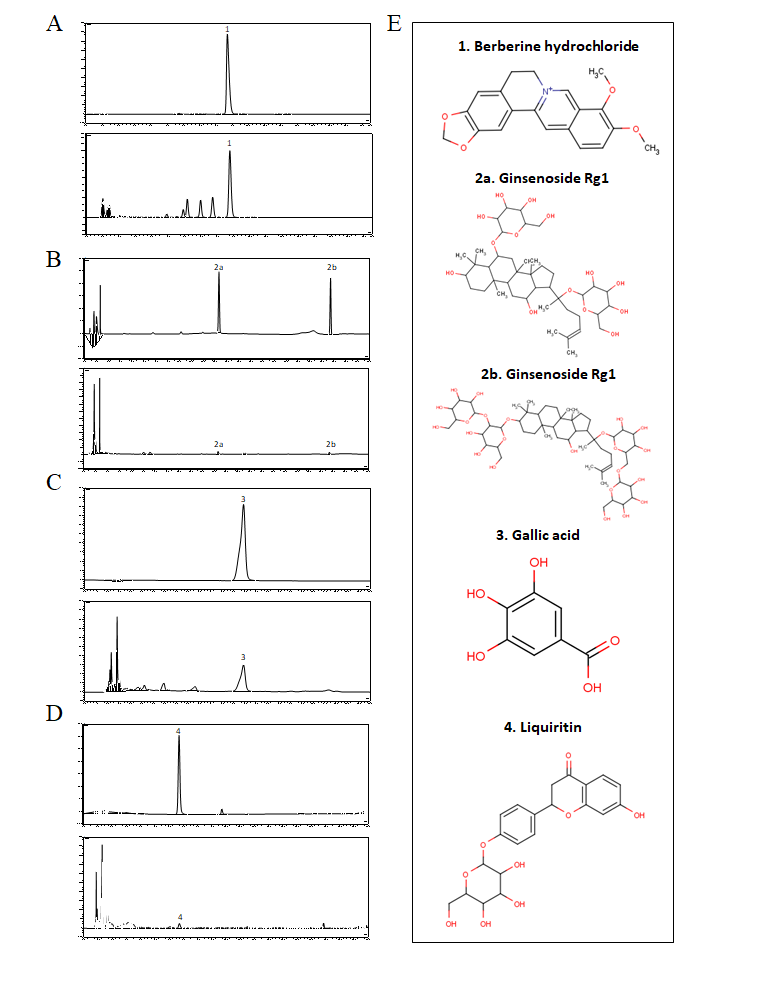


**Supplementary Figure S2. Microbial community functions predicted by PICRUSt using STAMP (version 2.1.3).**


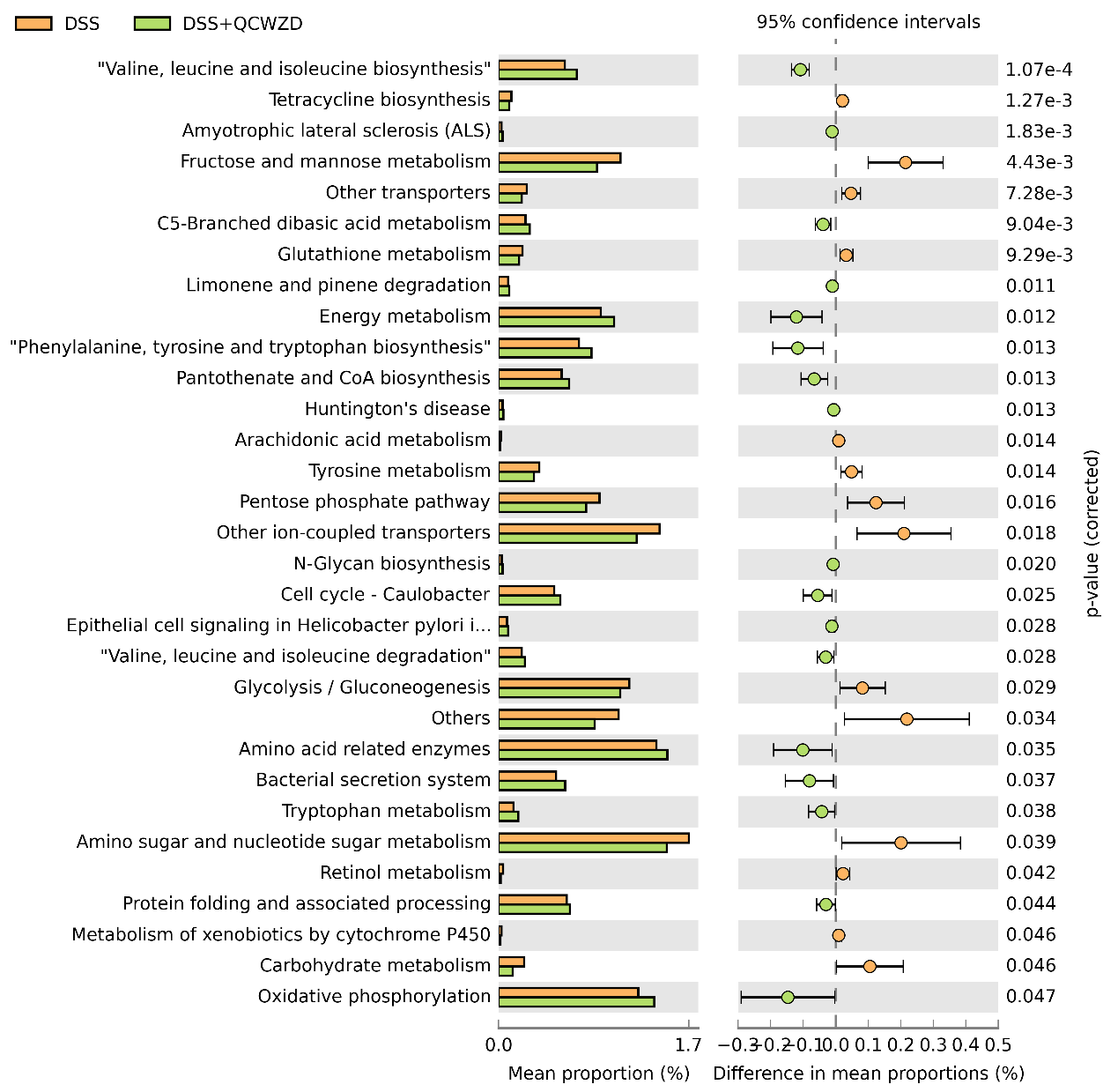

Supplement: Supplementary file 1 [file DataSheet1.DOCX]
